# Buffalo Medical and Surgical Association

**Published:** 1891-02

**Authors:** William H. Bergtold

**Affiliations:** Secretary


### BUFFALO MEDICAL AND SURGICAL ASSOCIATION.

Reported by WILLIAM H. BERGTOLD, M. D., Secretary.

The regular monthly meeting was held on Tuesday evening, November 4th, at 8.30, in the parlors of the Hotel Iroquois. The President, Dr. A. A. Hubbell, in the chair.

Present—Drs. Callahan, Wyckoff, Bartlett, Brodt, Jones, Thoma, Clark, Hartwig, Hubbell, Bayliss, Millring and Goltra.

The minutes of the previous meeting were read and, after corrections by Dr. Bartlett, were approved.

The name of Dr. Devan was proposed for membership. Dr. E. A. Smith was elected to full membership.

It was moved, seconded, and carried that the essayist's paper be postponed until the December meeting.

Under voluntary communications, Dr. M. Hartwig reported a case of abscess of the liver, in which he had adopted a novel method of treatment; the patient refused any appropriate operation, and the doctor then drained the abscess as follows: he thrust in a trocar and canula, pulled out the trocar, and in its place inserted a catheter, which fitted snugly to the canula, and withdrew the canula, leaving the catheter to act as a permanent drain.

A large amount of pus was evacuated at once, and more continued to appear for several days. The patient is now well on to recovery, though there is still a slight discharge. The tube was withdrawn and reinstated on several occasions after operation.

Dr. Bartlett thought that the idea and method were unique and novel. It seemed in this case to have been very satisfactory. He asked Dr. Hartwig what he considered some of these diagnostic signs of hepatic abscess, especially if incipient.

Dr. Bergtold thought that Dr. Hartwig was to be congratulated on his case, for he had met the indications very well.

Dr. Clark asked if Dr. Hartwig would use the same method in other cases.

Dr. Hartwig, in closing the debate, said that he himself was anxious about the motion of the liver displacing the drainage-tube, and in this case he believed that adhesions had formed which prevented any wide motions. The liver tissue, also, does not grasp a catheter as would muscle, and this is a danger in using this method. If he operated similarly again he would not empty the sac all at once, as he did this time. He left the tube in six days, and then gradually withdrew it.

Some characteristic symptoms of hepatic abscess he considers as follows:

1. Enlarged liver.
2. Localized pain.
3. Fever, may be variable; is more variable in multilocular abscesses.

Icterus is wanting in many cases. He mentioned acase where at the *post mortem* the liver was found to contain many minute abscesses, in the centre of each being a minute gall stone. In a normal liver we find an even line of dullness, while in abscesses or echinococcus this is irregular; finally, the last and one of our best diagnostic points is the use of the hypodermic needles, with which we should always search for pus; it never does any harm and is of very great usefulness.

Dr. Bartlett mentioned his method of treating dysentery by injections of bichloride 1-8000, using large and copious enemata. He reported, a number of years ago, about sixty cases treated according to this plan, and when he had good success.

His cases now number about 160, and he sees no reason for abandoning it. Children do not bear it so well, as we often have to chloroform them, in order to exhibit the enema. In order to be effectual the injections should be carried high up, while the patient lies on the right side, with the hips elevated. The effort should be to distend the color as fully as possible.

He has used the method with some cases of typhoid with good effect; here it acts both mechanically, in carrying away deleterious matters, as ptomaines, and antiseptically.

Dr. Hartwig thinks that in typhoid, these clysters do not reach the seat of disease, and that the whole alimentary tract could better be made aseptic by the use of calomel. He thinks, however, that the method is useful as an aid, and should be used when practicable.

Dr. Callahan said that he knew of a case of dysentery of eleven years' duration, which was cured by the use of bal. bichloride 1-8000 enemata.

